# A Cluster-based, Spatial-sampling Method for Assessing Household Healthcare Utilization Patterns in Resource-limited Settings

**DOI:** 10.1093/cid/ciaa1310

**Published:** 2020-12-01

**Authors:** Alexander T Yu, Rajani Shakya, Bikram Adhikari, Dipesh Tamrakar, Krista Vaidya, Stace Maples, Kashmira Date, Isaac I Bogoch, Caryn Bern, Farah Qamar, Mohammad T Yousafzai, Denise O Garrett, Ashley T Longley, Caitlin Hemlock, Stephen Luby, Kristen Aiemjoy, Jason R Andrews

**Affiliations:** 1 Division of Infectious Diseases and Geographic Medicine, School of Medicine, Stanford University, Stanford, California, USA; 2 Dhulikhel Hospital, Kathmandu University Hospital, Dhulikhel, Nepal; 3 Stanford Geospatial Center, Stanford University, Stanford, California, USA; 4 Global Immunization Division, Centers for Disease Control and Prevention, Atlanta, Georgia, USA; 5 Department of Medicine, University of Toronto, Toronto, Ontario, Canada; 6 Department of Epidemiology and Biostatistics, University of California, San Francisco, California, USA; 7 Aga Khan University, Karachi, Pakistan; 8 Applied Epidemiology, Sabin Vaccine Institute, Washington, DC, USA; 9 National Foundation for the Centers for Disease Control and Prevention, Atlanta, Georgia, USA

**Keywords:** healthcare utilization survey, SEAP, typhoid, cluster sampling, geospatial sampling

## Abstract

**Background:**

Implementation of population-based surveys is resource intensive and logistically demanding, especially in areas with rapidly changing demographics and incomplete or no enumeration of the underlying population and their residences. To remove the need for pre-enumeration and to simplify field logistics for the population healthcare utilization survey used for the Surveillance for Enteric Fever in Asia Project in Nepal, we incorporated a geographic information system–based geosurvey and field mapping system into a single-stage cluster sampling approach.

**Methods:**

A survey was administered to ascertain healthcare-seeking behavior in individuals with recent suspected enteric fever. Catchment areas were based on residential addresses of enteric fever patients using study facilities; clusters were randomly selected from digitally created grids using available satellite images and all households within clusters were offered enrollment. A tablet-compatible geosurvey and mapping system that allowed for data-syncing and use in areas without cellular data was created using the ArcGIS suite of software.

**Results:**

Between January 2017 and November 2018, we surveyed 25 521 households in Nepal (16 769 in urban Kathmandu and 8752 in periurban Kavrepalanchok), representing 84 202 individuals. Overall, the survey participation rate was 90.9%, with geographic heterogeneity in participation rates within each catchment area. Areas with higher average household wealth had lower participation rates.

**Conclusion:**

A geographic information system–based geosurvey and field mapping system allowed creation of a virtual household map at the same time as survey administration, enabling a single-stage cluster sampling method to assess healthcare utilization in Nepal for the Surveillance for Enteric Fever in Asia Project . This system removed the need for pre-enumeration of households in sampling areas, simplified logistics and could be replicated in future community surveys.

Burden of disease estimates for enteric fever, caused by *Salmonella enterica* serovars Typhi and Paratyphi, are important to help global, national, and subnational decision-makers prioritize vaccine rollouts, public health interventions, and resource allocation. Accurate population-level incidence estimates are sparse however, especially in resource-constrained settings [1, 2]. The Surveillance for Enteric Fever in Asia Project (SEAP) is a multicountry (Bangladesh, Nepal, and Pakistan) enteric fever study designed to help fill this gap. SEAP uses a hybrid surveillance method whereby facility-based incidence estimates are adjusted for community healthcare utilization. We describe the geographic cluster sampling methodology used in Nepal for the SEAP healthcare utilization survey.

Common approaches to assess enteric fever burden include population- and facility-based studies. Population-based studies prospectively follow a population cohort over time, repeatedly testing for enteric fever in anyone demonstrating compatible symptoms. These studies directly measure disease incidence in the population but have been sparse owing to the time and resources needed to administer [1, 3]. Facility-based surveillance studies are more common but have limited case capture rates because they only detect patients that seek care at study facilities, with a bias toward patients with more severe symptoms and ability to access care [3, 4]. A third approach, hybrid surveillance, uses a community healthcare utilization survey to understand the proportion of the population that would present to a study facility were they to experience symptoms consistent with enteric fever. This estimate of care seeking is then used to adjust enteric fever rates concurrently established at study facilities [3, 5].

Large community surveys such as healthcare utilization studies are often resource intensive with logistically demanding field implementation. They can be further complicated in areas with rapidly changing populations or with incomplete enumeration of the underlying population. In addition to these challenges, the Nepal site of SEAP included rural, low population-density study areas with difficult-to-traverse mountainous terrain. This made a pre-enumeration survey to list and map households, as performed at other SEAP country sites, time and cost prohibitive. Thus, we designed a geosurvey embedded in a geographic information system (GIS) field mapping system to allow real-time mapping and management of the healthcare utilization survey. By allowing research assistants to create a map in the field at the same time they administered the survey, this system removed the need for pre-enumeration and allowed continuous surveying over the study period.

## METHODS

Details of the overall SEAP methods, including the healthcare utilization survey, are described elsewhere in this supplement [6]. In brief, to adjust facility-based enteric fever rates for care-seeking behavior, a healthcare utilization survey was administered to households in each facility catchment area. Participation was voluntary and no compensation was provided. All consenting households were asked whether any members met a definition of suspected typhoid (fever lasting ≥3 days) in the past 8 weeks or had any hospitalization for febrile illness in the past year; household members meeting these criteria were administered a standardized questionnaire to ascertain healthcare-seeking behavior.

To determine households eligible for the survey, a single-stage geographic cluster sampling design was used. In Bangladesh and Pakistan, a pre-enumeration survey was conducted to first list and map households in selected clusters. In Nepal, instead of creating a pre-enumerated household map, the SEAP team created a map in the field at the same time as administration of the healthcare utilization survey using a geosurvey and GIS field mapping system. All households that had a front door within the boundaries of the cluster and had a consenting adult ≥18 years of age residing at the property for ≥6 months of the year were considered eligible. Families under the same roof were considered unique households if they had a separate kitchen. If household residents were not present at the time of the visit, the field team returned on 2 subsequent occasions to attempt enrollment.

### Study Site

**Figure 1.**
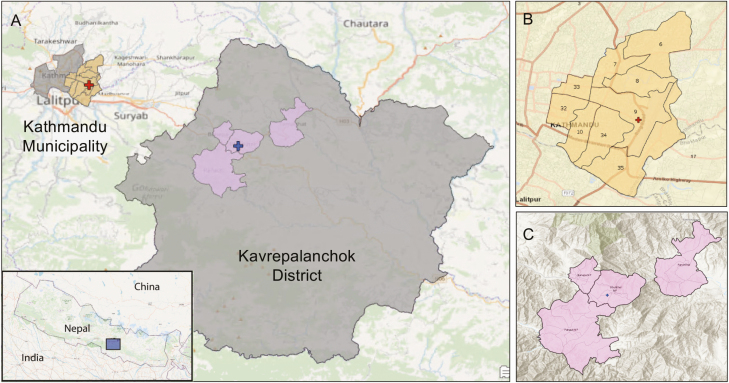
*A*, Map of urban Kathmandu Municipality and periurban Kavrepalanchok District, with inset showing location within Nepal. Nonstudy wards within Kathmandu Municipality and nonstudy municipalities within Kavrepalanchok District are shown in gray. *B*, Study catchment area of Kathmandu Municipality with catchment area wards in yellow. *C*, Study catchment area of Kavrepalanchok District. Red crosses in *A* and *B* represent Kathmandu Medical College; blue cross in *A,* Dhulikhel Hospital.

The Nepal SEAP site included 2 study facilities, Kathmandu Medical College and Teaching Hospital in Kathmandu Metropolitan City and Dhulikhel Hospital in Dhulikhel municipality in Kavrepalanchok District (Figure 1). Kathmandu is the capital city of Nepal and covers 20 square miles (49.45 sq km) with a density of 20 288 people per square km [7]. Similar to other large urban centers in developing regions, Kathmandu has experienced rapid population and housing growth, often overwhelming existing infrastructure. This situation was exacerbated by a magnitude 7.8 earthquake in 2015 that damaged or destroyed >800 000 buildings [8]. Kavrepalanchok District is a periurban region near the Kathmandu valley that covers 1396 square km of mountainous terrain, has a population density of 274 people per square km, and was also affected by the 2015 earthquake [7].

### Catchment Area

Study facilities were large, academic referral centers with inpatient and outpatient facilities, and existing laboratory infrastructure. Catchment areas were determined before the start of the study by mapping the home addresses of enteric fever or suspected enteric fever cases identified at study facilities. For Dhulikhel Hospital, the catchment area was determined from the most recent 100 enteric fever cases, as identified through SEAP phase I, a retrospective study on blood culture–confirmed enteric fever [9]. Kathmandu Medical College, which did not participate in the SEAP phase I study, used homes of the most recent 100 patients meeting SEAP phase II eligibility criteria (any of the following: blood culture positive for *S*. Typhi or Paratyphi, self-reported fever lasting ≥3 days, clinical suspicion or diagnosis of enteric fever, or pathognomonic ileal perforation). At both sites, administrative areas (wards or municipalities) representing 60% of cases were chosen as the catchment area (Figure 1). We selected administrative boundaries to simplify study eligibility assessment for patients presenting to the study site and reduce the risk of misclassification.

### Cluster Randomization

**Figure 2.**
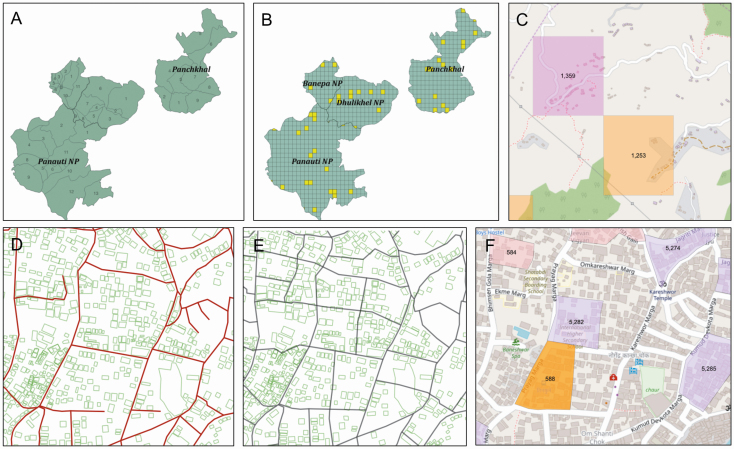
*A–C*, Kavrepalanchok District with labeled catchment municipalities and wards (*A*), with grid overlay and example random selection of grid squares chosen in yellow (*B*) and with zoomed view of 2 selected grids and their underlying house and road structure, using an OpenStreetMap base map (*C*). *D–F*, Kathmandu Municipality zoomed to 1:9000 scale, with underlying road infrastructure (*red*) and building footprints (*green*) outlined (*D*), road-based clusters constructed (*black outlines*) (*E*), and sample selected clusters (*purple and orange*) overlaid on an OpenStreetMap base map (*F*); *D*, *E*, and *F* all depict the same region. Numbers in (C) and (F) indicate assigned cluster study ID.

For Kavrepalanchok, a virtual grid consisting of rectangles with an area of 0.105 km^2^ (300 × 350 m^2^) each was created and overlaid on the catchment area map. These rectangles served as potential sampling clusters. Rectangles outside the catchment area were removed, and those at the boundaries were clipped to conform with ward boundary lines; in total, the grid consisted of 981 rectangles (Figure 2). In determining rectangle size, we balanced a desire to reduce clustering effects with efficiency tradeoffs related to travel times and low household density.

For the Kathmandu catchment area, rectangular clusters were not used because rectangular boundaries in dense, urban Kathmandu were difficult to delineate in the field and divided apartment buildings into multiple rectangles. Instead, clusters were created using roads as guidelines for boundaries (Figure 2). In total, >2500 street-based clusters were created. These clusters were tailored to approximate the same average area and designed to be small enough that the field team could complete each cluster within 2–3 days. The mean cluster area (standard deviation) was 0.0061 (0.0018) km^2^, an area equivalent to an 81 × 81-m^2^ square (Figure 2).

Once grids were created, we divided the population residing in each catchment area (2011 Nepal Demographic and Health Survey [7]) by the total number of clusters representing those areas to estimate the average number of households expected per cluster [10]. We used this number to calculate the number of clusters needed to reach sample size for each study facility catchment area, adjusted for expected intracluster correlation and resultant design effects [6]. We then took a simple random sample (nonweighted), using the sample function in R software, version 3.5.1.

### Field Surveying

**Figure 3.**
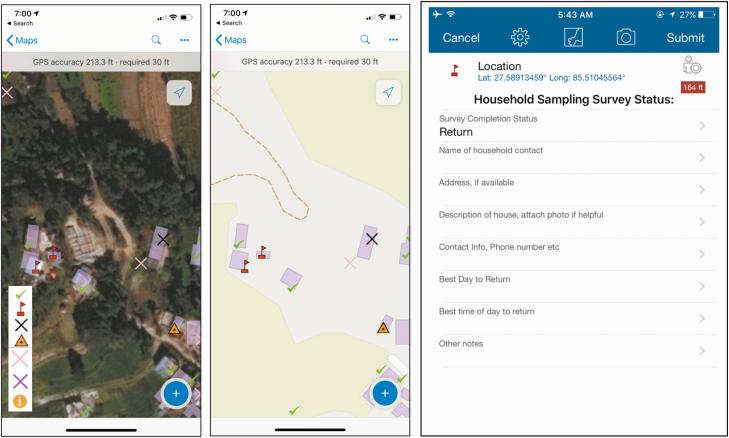
Tablet view of the geosurvey using World Imagery satellite (*left*) and World Street Map base map (*center*) base maps, with examples of successfully sampled houses (*green check mark*), houses with no one home and requiring a return visit (*red flag*), houses visited a second time but still requiring a third visit (*yellow triangle*), and houses declining the survey (*black X*). Geosurvey pins included questions and an option to directly attach photographs (*right*). Other labels and symbols include *i*, for general information, field, or administrative use; pink *X*, indicating not a house/skip; and purple *X,* indicating 3 attempts but no one home/do not return.

**Figure 4.**
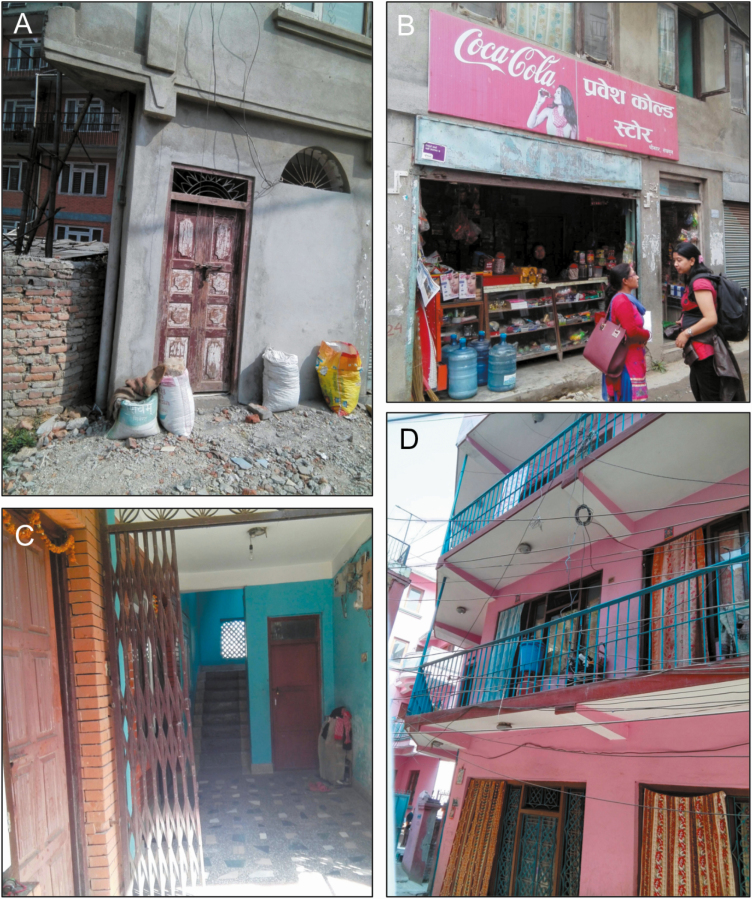
Photographs taken by field team to facilitate household identification on return visits, demonstrating doorway of a house in alleyway (*A*), residence behind a store (*B*), (C) household in a multifamily apartment (*C*), and a second-story apartment (*D*).

Research assistants were equipped with tablets loaded with Collector for ArcGIS (Environmental Systems Research Institute [ESRI] 2015; release 10.4) which displayed satellite imagery with superimposed outlines of clusters. As research assistants walked through clusters, they constructed virtual maps of the survey areas by approaching every visible structure within cluster boundaries to ascertain whether a household resided there, whether or not it appeared on the satellite images. They then labeled each structure using a geosurvey consisting of georeferenced pins. Pins identified whether structures were households, tracked household participation status, and included a set of questions and notes to facilitate return visits, if needed (eg, contact number and best time of day for a return visit) (Figure 3). Photographs could also be taken and attached to pins to help field staff find household during return visits (Figure 4).

This geosurvey was superimposed on GIS-based maps accessible to mobile devices in the field, and it was built using software from the ArcGIS platform (Collector for ArcGIS, ArcMap [2015; release 10.5] and ArcGIS Online [release December 2016]). Global positioning system (GPS) technology and cellular functionality were built into the tablets (Huawei Mediapad M2 tablets with an Android operating system) and could be used, when necessary, to help research assistants navigate the map.

**Figure 5.**
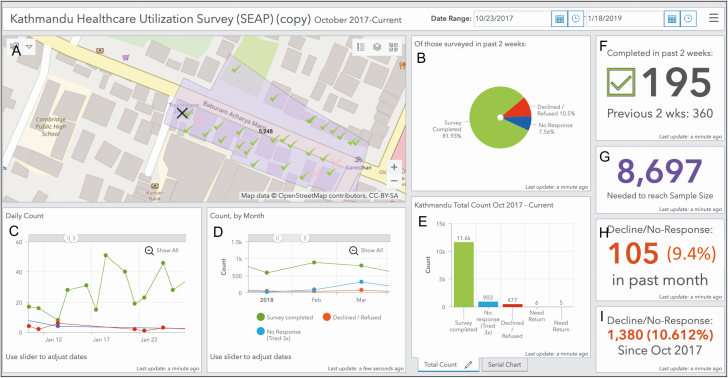
Real-time, Web-based, customizable dashboard for field monitoring. *A*, Map of Kathmandu catchment area with an example survey cluster in purple with geosurvey pins showing household survey status. *B*, Pie chart showing proportion of households surveyed in the past 2 weeks that have completed, declined, or could not be reached for the survey. *C–E*, Daily (*C*), monthly (*D*), and total (*E*) counts of completed, declined, and unreachable households. *F–I*, Additional indicators include a count of the number of households completed in the past 2 weeks compared with the prior 2-week period (*F*), household surveys needed to reach sample size (*G*), and decline rates over the past month (*H*) and since study initiation (*I*).

A supervisor created daily survey plans for research assistants and reviewed progress using ArcGIS Online. For tablets with access to mobile service, plans and progress automatically synced in real time between Collector and ArcGIS Online. For tablets without access to mobile service, syncing occurred when tablets were connected to wireless internet. This system allowed supervisors to monitor progress and make adjustments as needed. A real-time, online dashboard was also created using the Operations Dashboard for ArcGIS (ESRI 2016; release 10.3.4) in ArcGIS Online for monitoring of overall survey progress and efficiency (Figure 5). For quality assurance, a supervisor accompanied research assistants in the field at regular intervals to adherence to the assess study protocol. To monitor completeness of surveying within clusters, a supervisor independently visited a subset of clusters at regular intervals to look for households or buildings that may have been missed by research assistants. Data were periodically reviewed for completeness, and total number of households surveyed were routinely verified against collected household survey data and daily paper logs kept by surveyors.

### Assessing Participation Rate and Representativeness

Survey participation rate was calculated by dividing the number of households successfully surveyed by the total number of households identified in the sampling clusters. We then aimed to evaluate for potential biases in household participation, hypothesizing that wealthier households were less likely to participate in the study. To estimate wealth, we derived a household wealth index using a principal component analysis of assets [11] among households participating in the survey, including electricity and ownership of radio, television, landline telephone, mobile phone, computer, watch, bicycle, motorcycle, car, and bank account. However, we lacked data on households that declined to participate in the study. We therefore took 2 approaches to assess the relationship between neighborhood economic status and probability of participation in the study. First, we used an ordinary least-squares regression test to assess the relationship between survey participation rate and average household wealth, both at the cluster level. As a second approach, we performed spatial interpolation to estimate local densities of wealth and participation rates, using an inverse-distance weighted deterministic model.

Analyses of results from Kathmandu and Kavrepalanchok were independent of each other. Descriptive statistical analyses, χ ^2^ tests, *t* tests, and randomization were performed using R software, version 3.5.1. Grid, map, and geosurvey creation and spatial interpolation and regression tests were done using ArcMap software, version 10.5. Street and building metadata were derived from OpenStreetMap and the Humanitarian Data Exchange.

## RESULTS

Between January 2017 and November 2018, we approached 28 076 and enrolled 25 473 households (18 694 approached and 16 744 enrolled in Kathmandu, 9391 approached and 8729 enrolled in Kavrepalanchok). Enrolled households included 84 080 individuals (50 039 in Kathmandu and 34 041 in Kavrepalanchok). Households were enrolled from 712 clusters (420 in Kathmandu and 291 in Kavrepalanchok), with a median (interquartile range) of 32 (15–55) households enrolled per cluster in Kathmandu and 13 (5–29) per cluster in Kavrepalanchok. The survey was completed by 6 research assistants working 6 days a week; research assistants traveled to study areas in pairs for safety and transportation efficiency but surveyed households individually. On average per week, the team enrolled 248 households in Kathmandu or 277 households in Kavrepalanchok (6–8 households per day per research assistant).

Overall, the survey participation rate was 90.7% (25 473 of 28 085), lower in Kathmandu (89.6% [16 744 of 18 694]) than in Kavrepalanchok (93.0% [8729 of 9391]; *P* < .001 [*χ*^2^ test]). The rate of households declining participation was 3.7% (1050 of 28 085), higher in Kathmandu (4.7% [874 of 18 694]) than in Kavrepalanchok (1.9% [176 of 9391]; *P* < .001 [*χ*^2^ test]). The proportion of households not home and thus unavailable to survey was 5.6% (1562 of 28 085), slightly higher in Kathmandu (5.8% [1090 of 18 694]) than in Kavrepalanchok (5.0% [472 of 9391]; *P* < .05 [*χ*^2^ test]). Healthcare utilization and other results from the survey are reported separately in this supplement [6].

**Figure 6.**
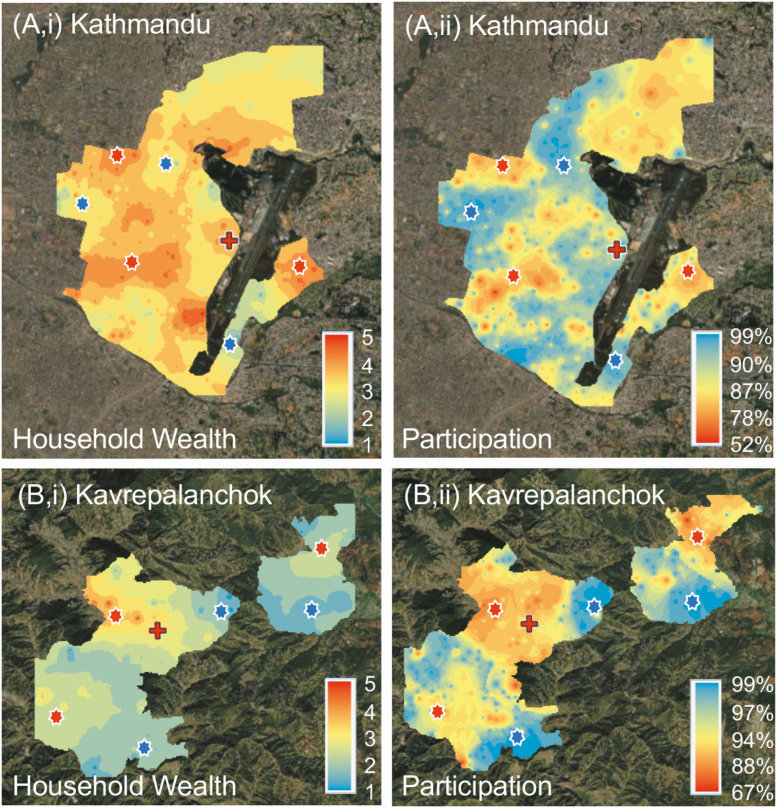
Maps of Kathmandu (*top*) and Kavrepalanchok (*bottom*) catchment areas showing average household wealth index (quintile, with 5 the wealthiest) (*top and bottom lef*t) and survey participation rate (*top and bottom right*). Areas with higher wealth indices were correlated with lower survey participation rates (*red asterisks*), and areas with lower household wealth indices with higher survey response rates (*blue asterisks*). Red crosses note location of study facility.

The household wealth quintile was higher in Kathmandu (mean [standard deviation], 3.1 [1.3]) than in Kavrepalanchok (2.3 [1.4]; *P* < .001 [*t* test]). Spatial interpolations demonstrated geographic heterogeneity in participation rate and wealth quintile. Areas with higher average wealth quintile tended to correlate with lower participation rates (Figure 6 and Supplementary Figure 1). From the regression model, for every quintile increase in wealth, the participation rate decreased by 5.15% (95% confidence interval, 3.63%–6.66%; *P* < .001) in Kathmandu and 3.12% (1.54%–4.70%; *P* < .001) in Kavrepalanchok. The distance to the study hospital was not correlated with participation. Additional analyses of spatial risk factors associated with household wealth, fever, care seeking, water source and distance to facility are described elsewhere in this supplement [12].

## DISCUSSION

Conducting population-based household surveys in resource-constrained environments poses a number of logistical challenges, particularly when detailed infrastructure maps are out of date and prior enumeration of households is not available. We incorporated a geosurvey into a cluster random sampling scheme to survey >25 000 households in Nepal without pre-enumeration data. We found that this approach was easy to operationalize and enabled an efficient survey of households in both dense urban areas as well as rural settings.

Pre-enumeration can be as time and resource consuming as the survey itself and is susceptible to population or infrastructure changes, especially if delays occur between pre-enumeration and surveying. These drawbacks can offset statistical efficiency gains from the process. The main reasons to pre-enumerate are to enable randomized sample selection, create a field map for surveyors to locate households, and generate a list to track progress. Our geosurvey allowed research assistants to create a dynamic, up-to-date virtual map of all buildings encountered in the field. All households encountered could be surveyed on the same visit; if not available to answer the survey, households could be geotagged for a another visit, and maps could be updated if any changes in the neighborhood were encountered during return visits. These features, embedded in a cluster sampling method, allowed us to achieve the same benefits gained from pre-enumeration with reduced time and resource costs. It also enabled us to continuously sample throughout the study period, reducing worry of underlying population or infrastructure changes, and provided a means to verify completeness in the field and track progress in real time.

A variety of sampling methods have been used for geospatially randomized community surveys when a pre-enumerated list of households is not available. In the Typhoid Fever Surveillance in Africa Program study, randomization was achieved by selecting from a digital map of building footprints identified via satellite imagery [13, 14]. The validity of this method is dependent on accurate satellite images and building footprint data. If building footprint data are unavailable, as was the case in the Typhoid Fever Surveillance in Africa Program study and in many parts of the world, then they have to be manually created. Generally, this method also assumes that each building represents a single family household. Another method, used in one of the original descriptions of hybrid surveillance, took patients with recently diagnosed enteric fever as starting points in the community; the next 5 closest households were skipped and the sixth household was surveyed [5]. This type of sampling can be susceptible to selection bias in the field. Another strategy is to randomly select geospatial coordinates to survey [15]. In areas with mixed population density, this method can oversample lower-density areas and can lead to selection bias if multiple households live at the same location, such as in an apartment building.

Our strategy did not exempt us from problems common to other household survey strategies in resource-limited settings. In urban Kathmandu, we noted informal housing in hidden yards, behind gates, in abandoned homes and in construction areas. We also found individuals residing in their places of work, including storefronts, offices and employer homes. Although we surveyed all informal housing we could find, these types of households are easily missed even with intense scrutiny. Informal housing is also at risk of being missed if outdated satellite images are used and areas that appear to have no buildings are not verified in the field. Because informal households are more likely to be missed than formal households, survey results could underrepresent lower socioeconomic status households, a population associated with higher risk for many diseases and least likely to seek care [16, 17].

At the same time, we found evidence that households in wealthier neighborhoods were less likely to participate in the study. Research assistants anecdotally reported that in wealthier neighborhoods, households were more likely to have barriers preventing access to the building and more likely to decline to participate in the study; in addition, research assistants reported a greater perception of mistrust of strangers in these neighborhoods and increased chance of nonresponse at the door even when a resident was visibly present. Our survey participation results supported this theory, with wealthier neighborhoods having lower participation rates and conversely, areas with lower wealth demonstrating higher participation rates (Figure 6 and Supplementary Figure 1). This finding is limited, however, because houses that did not participate could not provide wealth data, and thus, our analysis relied on data aggregated at the cluster level. Because care-seeking behaviors and disease risk are often associated with household wealth, quantifying participation biases in these surveys may enable adjustments to generate more accurate and representative estimates of care seeking or disease burden.

We used the ArcGIS suite of software to build our system, which was simple, reliable, but not free (cost as of 2020: $700 for an annual subscription, including all licenses used in this study). A similar system could be designed using open-source software, though at the time of study initiation we were unable to find customizable software allowing both real-time syncing and an offline option. Because households are identified in the field, recent or high-resolution satellite or aerial imagery is not a requisite for this method. However, updated imagery can help identify housing otherwise obscured in the field by buildings or terrain. Low-resolution imagery can also compound problems related to GPS inaccuracy, making it more difficult to find houses, track progress, and accurately geotag data. To prepare research assistants for potential imagery, GPS, and cellular service limitations, we spent multiple days training exclusively on map reading and navigation without GPS or internet connectivity and designed exercises in which research assistants worked to find practice houses in the field.

Geographic cluster sampling can be an effective way to implement community surveys. Incorporation of a geosurvey for the field operationalization of the healthcare utilization survey in the Nepal site of SEAP removed the need to pre-enumerate households, allowed continuous sampling, and simplified field logistics by making planning and oversight easier. This method benefits from but is not reliant on high-resolution, recent satellite or aerial imagery, and future efforts will likely be able to replicate these processes using open-source software. GIS-based mapping systems for the operationalization of cluster sampling can be an effective and resource-saving method for implementing healthcare utilization or other household surveys in the future.

## Supplementary Data

Supplementary materials are available at *Clinical Infectious Diseases* online. Consisting of data provided by the authors to benefit the reader, the posted materials are not copyedited and are the sole responsibility of the authors, so questions or comments should be addressed to the corresponding author.

ciaa1310_suppl_Supplementary_Figure_1

## Notes

***Acknowledgments.*** The authors want to thank all of the research assistants and staff who helped make this work possible.

***Financial support.*** This work was supported by the Bill & Melinda Gates Foundation (grants NV-000572 and OPP1113007 and grants to S. L. and J. R. A.), INV-008335.

***Supplement sponsorship.*** This supplement is sponsored by the Sabin Vaccine Institute and made possible by a grant from the Bill & Melinda Gates Foundation.

***Potential conflicts of interest.*** K. D. and A. T. L. disclose that the CDC Foundation has a conditional gift agreement with the Sabin Vaccine Institute (primary grantee for the Surveillance for Enteric Fever in Asia Project grant from the Bill & Melinda Gates Foundation). All other authors report no potential conflicts. All authors have submitted the ICMJE Form for Disclosure of Potential Conflicts of Interest. Conflicts that the editors consider relevant to the content of the manuscript have been disclosed.
