# Supplementary figures and images for: A Cluster-based, Spatial-sampling Method for Assessing Household Healthcare Utilization Patterns in Resource-limited Settings
Source: Clin Infect Dis. 2020 Dec 1;71(Suppl 3):S239–47. doi: 10.1093/cid/ciaa1310 (PMC7705878; doi:10.1093/cid/ciaa1310)

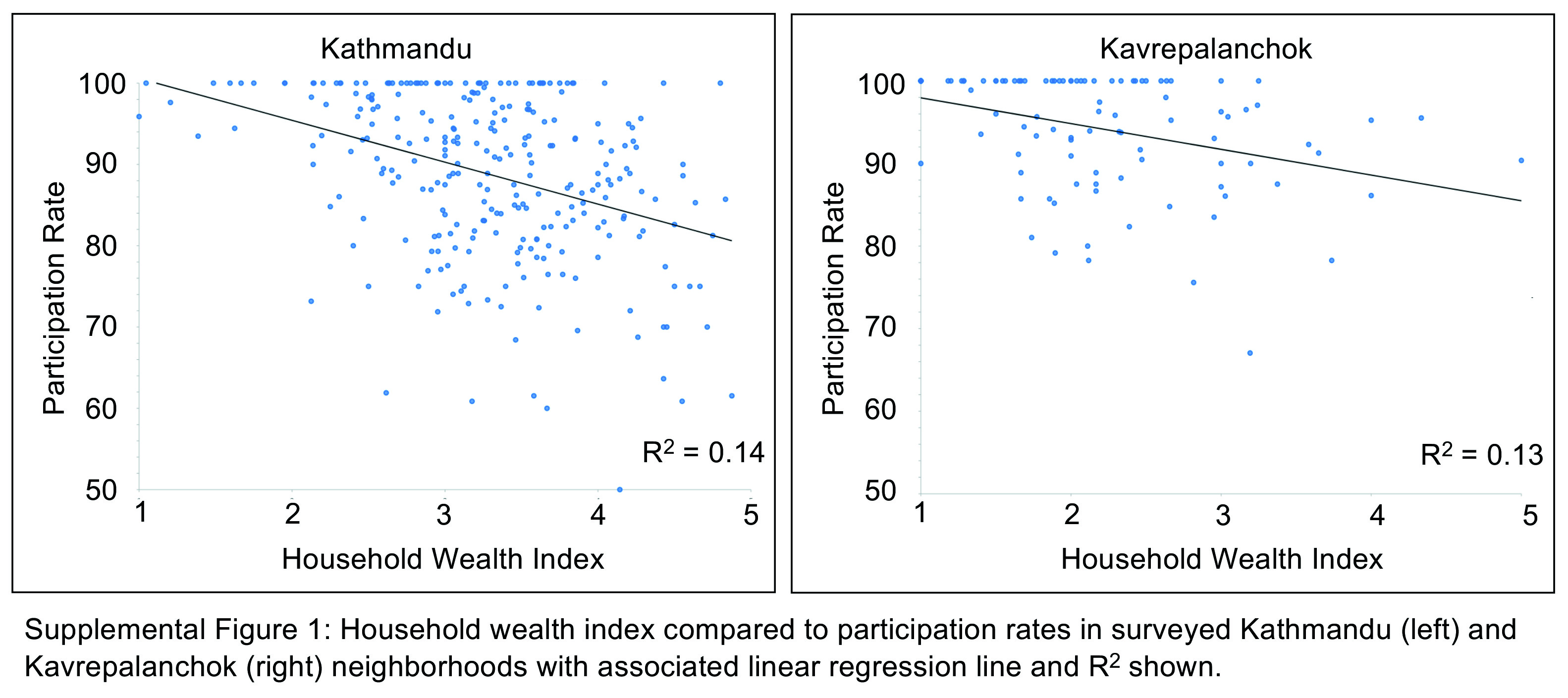

Supplement: ciaa1310_suppl_Supplementary_Figure_1 [file ciaa1310_suppl_supplementary_figure_1.jpeg]
